# Towards an understanding of the molecular basis of effective RNAi against a global insect pest, the whitefly *Bemisia tabaci*

**DOI:** 10.1016/j.ibmb.2017.07.005

**Published:** 2017-09

**Authors:** Yuan Luo, Qingguo Chen, Junbo Luan, Seung Ho Chung, Joyce Van Eck, R. Turgeon, Angela E. Douglas

**Affiliations:** a Department of Entomology, Cornell University, Ithaca, NY 14853, USA; b Plant Biology Section, School of Integrative Plant Science, Cornell University, Ithaca, NY 14853, USA; c Boyce Thompson Institute, Ithaca, NY 14853, USA; d Department of Molecular Biology & Genetics, Cornell University, Ithaca, NY 14853, USA

**Keywords:** *Bemisia tabaci*, dsRNA degradation, RNA interference, RNAi efficacy

## Abstract

*In planta* RNAi against essential insect genes offers a promising route to control insect crop pests, but is constrained for many insect groups, notably phloem sap-feeding hemipterans, by poor RNAi efficacy. This study conducted on the phloem-feeding whitefly *Bemisia tabaci* reared on tomato plants investigated the causes of low RNAi efficacy and routes to ameliorate the problem. Experiments using tomato transgenic lines containing ds-*GFP* (green fluorescent protein) revealed that full-length dsRNA is phloem-mobile, ingested by the insects, and degraded in the insect. We identified *B. tabaci* homologs of nuclease genes (*dsRNase*s) in other insects that degrade dsRNA, and demonstrated that degradation of ds-*GFP* in *B. tabaci* is suppressed by administration of dsRNA against these genes. dsRNA against the nuclease genes was co-administered with dsRNA against two insect genes, an aquaporin *AQP1* and sucrase *SUC1*, that are predicted to protect *B. tabaci* against osmotic collapse. When dsRNA constructs for *AQP1, SUC1, dsRNase1* and *dsRNase2* were stacked, insect mortality was significantly elevated to 50% over 6 days on artificial diets. This effect was accompanied by significant reduction in gene expression of the target genes in surviving diet-fed insects. This study offers proof-of-principle that the efficacy of RNAi against insect pests can be enhanced by using dsRNA to suppress the activity of RNAi-suppressing nuclease genes, especially where multiple genes with related physiological function but different molecular function are targeted.

## Highlights

• Tomato plants transformed with dsRNA deliver full-length dsRNA to the phloem-feeding whitefly *Bemisia tabaci*.
• Orally-acquired dsRNA is degraded in the body of the whitefly *B. tabaci*.
• The *B. tabaci* genome encodes three non-specific nucleases, two of which are expressed in the gut.
• RNAi-mediated suppression of nuclease expression reduces degradation of other dsRNA molecules ingested by whiteflies.
• Whitefly mortality on diets is enhanced by stacking oral RNAi against insect osmoregulation genes and nucleases.

## Graphical abstract

**Figure:**
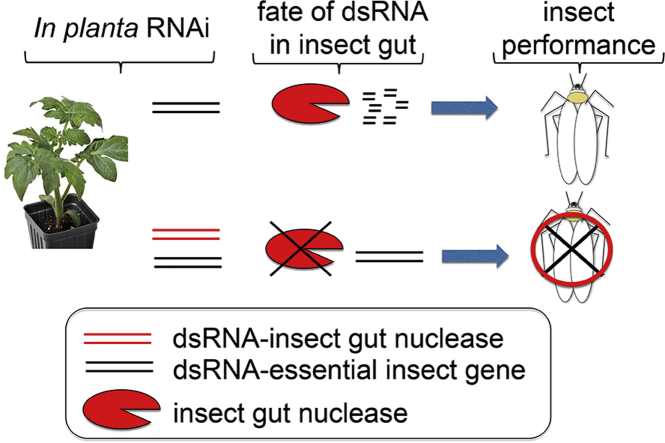

## 1 Introduction

RNA interference (RNAi) holds great promise as a novel strategy against insect pests of agricultural crops (Scott et al., 2013, Whyard et al., 2009, Xue et al., 2012). This is because, in principle, the only information needed to target an essential insect gene with exquisite specificity is the insect gene sequence; and the double-stranded RNA molecules (dsRNA) can be delivered via transgenesis of the crop plant. Specifically, the plant is engineered to express dsRNA against the insect gene of interest. On ingestion by the insect, the dsRNA is internalized into cells, where it is cleaved by an insect dsRNA-specific enzyme, Dicer-2, into small interfering RNA (siRNA, ca. 21 nt) that guides the Argonaute protein of the RNA induced silencing complex (RISC) to degrade complementary mRNAs (Wilson and Doudna, 2013, Zamore et al., 2000). *In planta* RNAi has yielded significant plant protection against the western corn rootworm *Diabrotica virgifera virgifera* (Baum et al., 2007), cotton bollworm *Helicoverpa armigera* (Mao et al., 2007) and Colorado potato beetle *Leptinotarsa decemlineata* (Zhang et al., 2015), and insecticidal RNAi in transgenic crops are reported to be near commercial release.

Despite these substantial advances, many RNAi studies on insects have yielded moderate or variable knock-down of gene expression, with limited effects on insect phenotype and performance (Belles, 2010, Scott et al., 2013, Terenius et al., 2011). These problems apply particularly to plant sap-feeding insects, such as whiteflies, aphids, psyllids and planthoppers, including major pests and vectors of plant viruses, where RNAi against essential genes often reduces growth or reproduction, but has small or no effect on survivorship (Coleman et al., 2015, Pitino et al., 2011, Thakur et al., 2014, Tzin et al., 2015, Zha et al., 2011).

A possible cause of the limited efficacy of *in planta* RNAi against many insects is that the dsRNA molecules ingested by the insects may be delivered poorly to the intracellular RNAi machinery in the insect cells. The plants are engineered to produce long dsRNA molecules, but the dsRNA can be processed by the plant RNAi machinery to generate small RNAs (sRNAs, 21–24 nt) (Baulcombe, 2015, Melnyk et al., 2011b). It is widely believed that sRNAs are less effective than long dsRNA molecules as triggers of insect RNAi (Huvenne and Smagghe, 2010). However, this view is based largely on studies of RNAi in cultured insect cells (Saleh et al., 2006, Ulvila et al., 2006), and appears to be contradicted by a study on the phloem-feeding aphid *Myzus persicae* that obtained similar gene expression reduction with dsRNA and diced dsRNA (Mutti et al., 2006). Further possible impediments to dsRNA delivery are, first, that the plant dsRNA can be degraded by non-specific nucleases in the insect saliva, gut lumen or hemolymph (Allen and Walker, 2012, Christiaens et al., 2014, Garbutt et al., 2013, Liu et al., 2012b, Luo et al., 2013, Spit et al., 2017, Wang et al., 2016, Wynant et al., 2014), and, second, that the intracellular RNAi machinery in the insect may have low activity (Garbutt and Reynolds, 2012).

The purpose of this study was to identify the factors in the plant and insect that limit the efficacy of RNAi against phloem-feeding insects, and to use this information for improved design of RNAi. Our experiments were conducted on the whitefly *Bemisia tabaci*, which is a globally-important pest of many crops (Navas-Castillo et al., 2011). As in various other insects (see above), multiple RNAi studies of *B. tabaci* have yielded incomplete knock-down of gene expression (Ghanim et al., 2007, Luan et al., 2013, Raza et al., 2016, Thakur et al., 2014, Upadhyay et al., 2011), constraining the application of RNAi as a control strategy for this pest.

We hypothesized that whitefly RNAi may be limited by processing of dsRNA to small RNAs (sRNAs) in the plant and, additionally or alternatively, by nonspecific degradation of dsRNA in the insect. We tested these hypotheses by following the fate of dsRNA constructed against a 370 nt fragment of the green fluorescent protein (*GFP*) gene of the jellyfish *Aequorea victoria*, administered to the insects via artificial diets and plant transgenesis. These experiments led us to identify non-specific degradation of dsRNA by *B. tabaci*, which we then reduced by RNAi against two candidate *B. tabaci* nuclease genes. In our final experiments, we tested the efficacy of stacking RNAi against the nuclease genes with RNAi against candidate essential genes of *B. tabaci*. Our genes of choice were an aquaporin and a glucohydrolase of family GH-13, previously identified as candidate osmoregulation genes that protect the insect from rapid dehydration and death (Jing et al., 2016, Mathew et al., 2011). Following the nomenclature of Jing et al. (2016), we call these genes *BtAQP1* and *BtSUC1*, respectively. This study yields new insights into the fate of dsRNA administered to phloem-feeding insects by *in planta* RNAi, and the value of this technology as a novel insect pest control strategy.

## 2 Material and methods

### 2.1 Plants and insects

Tomato plants (*Solanum lycopersicum* cv. Florida Lanai) (seeds provided by C. L. McKenzie, USDA-ARS Fort Pierce, FL, USA) were grown in compost supplemented with Miracle-Gro^®^ Water Soluble All Purpose Plant Food in climate-controlled chambers at 25 ± 2 °C with a 14L:10D light cycle at 400 μmol m^−2^ s^−1^ PAR.

The *Bemisia tabaci* MEAM1 culture (mtCO1 GenBank accession no. KM507785) was derived from a collection from poinsettia (*Euphorbia pulcherrima* Willd. Ex Klotzsch) in Ithaca, NY, USA in 1989. The insects were maintained on 5-6-week-old tomato plants at 25 ± 2 °C with a 14L:10D light cycle at 400 μmol m^−2^ s^−1^ PAR. One-day-old adult males and female insects were caged to test plant leaves using BugDorm-1 Insect Cages (Bio Quip, Rancho Dominguez, CA) or custom clip-cages, and fed on a sterile artificial liquid diet (Douglas et al., 2001) containing 0.5 M sucrose and 0.15 M amino acids in Parafilm sachets. Some experiments used isolated guts, dissected with fine pins from adult female and male insects into phosphate-buffered saline.

### 2.2 RNA extractions

For total RNA isolation, leaves or whiteflies were homogenized in TRIzol^®^ Reagent (Cat# 15596-026, Thermo Fisher Scientific, Waltham, USA) with 1:1 (vol) Lysing Matrix D Bulk beads (Cat# 116540434, MP Biomedicals, Santa Ana, USA) on a MP FastPrep-24™ homogenizer (Cat# 116004500, MP Biomedicals, Santa Ana, USA) with 5.5 M/S for 2 × 30 s. The lysed samples were centrifuged at 13,000 rpm for 3 min to remove the beads and RNA was isolated from the supernatant following the manufacturer's instructions. LMW (low molecular weight) and HMW (high molecular weight) RNA isolation employed the same protocol as above, except that the supernatant of the lysate homogenate was mixed with 70% ethanol (1:1, by vol) and passed through a RNeasy mini column (Cat# 74104, Qiagen, Venlo, Limburg, USA). RNAs longer than 200 nt were collected in the column and washed from the column following the RNeasy mini kit protocol. RNAs shorter than 200 nt were precipitated from the flowthrough with 100% isopropanol (1:1, by vol) and 3 M sodium acetate (pH 5.2, 10:1, by vol), followed by 75% ethanol wash and then dissolved in nuclease-free water. RNA samples used for qRT-PCR were further processed to remove genomic DNA by incubation with 10 μl DNase 1, using the reagents and protocol in the RNase-free DNase set (Cat# 79254, Qiagen, Valencia, USA). To recover dsRNA from diets, samples of the diet were combined with isopropanol (1:1, by vol) and 3 M sodium acetate pH 5.2 (1:10 by vol). The concentration of RNA was determined spectrophotometrically with a Nanodrop-2000 (Thermo Fisher Scientific, Waltham, USA), and RNA integrity was verified by denaturing formaldehyde gel-electrophoresis.

### 2.3 Synthesis of cDNA and dsRNA

cDNA libraries for amplification of whitefly genes were prepared with oligo (dT) primers using the Reverse Transcription System (Cat# A3500, Promega, Madison, USA) following the manufacturer's instructions. Candidate osmoregulatory genes, comprising aquaporin *AQP1* (NCBI Accession KX390870) (Jing et al., 2016, Mathew et al., 2011), sucrase *SUC1* (also known as α-glucohydrolase *GH13-1* (Jing et al., 2016)) (NCBI Accession KX390872), and candidate nuclease genes *dsRNase1* (NCBI Accession KX390873) and *dsRNase2* (NCBI Accession KX390874) identified in this study, were amplified from the cDNA template in a reaction mix containing 0.4 μM primers (Table S1), 2 U Invitrogen Platinum Taq DNA polymerase (Cat# 10966018, Thermo Fisher Scientific, Waltham, USA), 1.5 mM MgCl_2_ and 200 ng cDNA template in 25 μl volume, using a Techne thermal cycler. The thermal profile comprised 2 min at 94 °C for initial denaturation, 30 cycles with 95 °C for 30 s, 55 °C for 30 s, 72 °C for 1 min (depending on products length, 1 kb min^−1^) and final extension cycle of 72 °C for 5 min. Amplicon sequences were verified by Sanger sequencing, then introduced into _P_GEM-T vector (Cat# A1360, Promega, Madison, USA) and transformed into DH5α™ competent cells. The plasmid was extracted and, following confirmation by sequencing, used as template for dsRNA synthesis with primers listed in Table S1.

The dsRNA was synthesized using the AmpliScribe ™ T7-Flash Transcription Kit (Cat# ASF3257, Epicentre Biotechnologies, Madison, USA), according to the manufacturer's instructions. The templates were the pGFP2 plasmid for ds-*GFP*, and plasmids obtained for the whitefly genes identified above. The dsRNA product was quantified by Nanodrop, and run on a gel with 1 kb plus molecular weight ladder (Cat# 10787026, Thermo Fisher Scientific, Waltham, USA) to confirm the predicted size.

### 2.4 Construction of dsRNA expression cassette

The dsRNA expression cassette was constructed in the pHANNIBAL vector. A 370 nt *GFP* sequence was amplified and inserted in inverted orientation into pHANNIBAL using the PDK intron as a spacer and different restriction sites, *Xho*I and *Eco*RI for sense *GFP* and *Hin*dIII and *Xba*I for the inverted *GFP* sequence. Two phloem-companion cell-specific promoters *Galactinol Synthase* from melon *Cucumis melo, CmGAS1* (Ayre et al., 2003, Haritatos et al., 2000) and sucrose-H^+^ symporter from *Arabidopsis thaliana*, *AtSUC2* (Dunoyer et al., 2007, Truernit and Sauer, 1995) were cloned and inserted into the binary vector pER8 using *Xho*I/*Spe*I and *Xho*I restriction sites separately. The *GFP*-intron-r*GFP* cassette was assembled using the Gibson assembly kit (New England Biolabs Cat# E2611S) following manufacturer's instructions, and inserted downstream of the promoter in pER8 vector using a *Xho*I restriction site for *AtSUC2* and *Spe*I site for *CmGAS1*.

### 2.5 Generation of tomato transgenics with dsRNA gene construct

The binary vector pER8 was introduced via electroporation into *Agrobacterium tumefaciens* strain LBA4404. Kanamycin selection at 50 μg ml^−1^ was used to select for transformants. Transformation of the tomato plants was performed according to methods described by Van Eck et al. (2006). Putative transgenic plants were transferred to soil and maintained in an incubator at 25 ± 2 °C with a relative humidity of 60–70% and with a 14L:10D light cycle at 400 μmol m^−2^ s^−1^ PAR. Total DNA was extracted from leaves of 5-6-week-old transgenic plants and verified for transformation by PCR with sequence specific primers (Table S1). Eight transgenic lines were confirmed for each promoter.

### 2.6 Administration of dsRNA to whiteflies

dsRNA was administered to adult insects either via artificial diets (at 0.1–1 μg μl^−1^, varying with experiment with full details provided in the relevant figure legends) or via transgenic tomato lines. For analysis of the fate of ds-*GFP*, 100 whiteflies were administered to each diet cage and ca. 250 whiteflies were caged to each plant. For insect performance experiments on diets, 10 replicate groups of 40 adult whiteflies (one day post-emergence) were applied to each diet treatment and mortality was monitored daily over 6 days, with insects transferred to fresh diet containing dsRNA every two days. At the end of each experiment, all live insects were transferred to 500 μl TRIzol^®^ Reagent (Cat# 15596-026, Thermo Fisher Scientific, Waltham, USA) and stored at −80 °C prior to isolation of total RNA (as above).

### 2.7 Northern blots

RNA extracted from whiteflies and plants was separated on denatured polyacrylamide-urea gels (SequaGel - UreaGel System, cat# EC-833, National Diagnostics, Atlanta, GA, USA) containing 8% monomers. The gel was pre-run at 250 V in 0.5 × TBE buffer for 30 min, then 20 μg sample RNA was combined with an equal volume of Gel Loading Buffer II (Cat# AM8547, Thermo Fisher Scientific, Waltham, USA) and heated at 95 °C for 4 min to denature the RNA. Samples were loaded in urea-cleaned wells and run at 250 V in 0.5 × TBE buffer until the loading dye migrated to the far end of the gel. Uniform sample loading was confirmed by staining of 5S rRNA with SYBR Gold, followed by transfer to Hybond-NX membrane (Cat# RPN 203T, GE Healthcare, Wilkes-Barre, PA) with the Owl™ HEP Series Semidry Electroblotting Systems at 0.4 A for 1 h. The transferred RNA was cross-linked using a UV crosslinker at 120 kJ for 30 s for HMW RNA and by a published procedure (Pall and Hamilton, 2008) for LMW RNA.

The 370 nt *GFP* antisense probe used for northern blotting was generated using the MAXIscript^®^ *In Vitro* Transcription Kit (Cat# AM1308, Thermo Fisher Scientific, Waltham, USA) with 3.125 μM alpha-^32^P UTP (10 mCi ml^−1^, 800 Ci mmol^−1^, Perkin Elmer, Waltham, USA), 5 μM UTP and 100 μM ADP. Unincorporated ^32^P-label was removed using a RNeasy mini column (Cat# 74104, Qiagen, Venlo, Limburg, USA). The ^32^P-labeled probe was brought to 100 μl with nuclease-free water, mixed with 350 μl buffer RLT (RNeasy mini kit, Cat# 74104, Qiagen, Venlo, Limburg, USA) and 250 μl 100% ethanol, collected onto a RNeasy mini column, washed twice with buffer RPE (from RNeasy mini kit, Cat# 74104, Qiagen, Venlo, Limburg, USA), and eluted with 40 μl nuclease-free water. For each membrane, half of the probe was heated at 95 °C for 4 min and immediately added to the pre-hybridized membrane at 2 × 10^6^ cpm ml^−1^ final concentration. For HMW RNA detection, pre-hybridization and hybridization were conducted at 68 °C using Ambion Ultrahyb buffer (CAT# AM8670, Thermo Fisher Scientific, Waltham, USA) according to the manufacturer's protocol. The blot was washed twice in 2× SSC, 0.1% SDS buffer at 68 °C, followed by two washes in 0.1× SSC, 0.1% SDS buffer at the same temperature. For LMW RNA detection, the blot was pre-hybridized in hybridization buffer (5 × SSC, 20 mM Na_2_HPO_4_ (pH 7.2), 7% SDS, 2 × Derhardt's solution) at 50 °C for at least 2 h, then hybridized with a final concentration of 2.5 × 10^6^ cpm ml^−1^ probe in the same buffer at 50 °C overnight. The membrane was washed four times at 50 °C in non-stringent wash buffer (3× SSC, 25 mM NaH_2_PO_4_ pH 7.5, 5% SDS) and once in stringent wash buffer (1× SSC, 0.1% SDS), and then exposed for autoradiography. The signal was collected on phosphor screen (Molecular Dynamics) and scanned using a Typhoon 9400 fluorescent imager.

ImageJ was used for ds-*GFP* band density analyses.

### 2.8 qRT-PCR

To quantify the expression of target whitefly genes, qRT-PCR was performed with RNA extracted from three biological replicates of whiteflies. cDNA was prepared using random primers of High-Capacity cDNA Reverse Transcription Kit or SuperScript™ II Reverse Transcriptase (Cat# 4368814 and 18064014, Thermo Fisher Scientific, Waltham, USA) following the manufacturer's instructions. For qRT-PCR, the 20 μl reaction mix comprised 10 μl Master Mix (Bio-Rad, Hercules, CA) or Power SYBR Green PCR Master Mix (Applied Biosystems, Carlberg, CA, USA), precisely 1 μl cDNA template and 0.5–2 μl 10 μM primers qRT-PCR primers (Table S1) designed with Primer Premier 5.0 software (Premier Biosoft International, Palo Alto, CA). Amplifications were conducted in a C1000TM Thermal cycler (Bio-Rad, Hercules, CA) with the following thermal profile: 95 °C for 5 min, 40 amplification cycles of 95 °C for 15 s, 55 °C for 30 s, and dissociation cycle of 95 °C for 15 s, 55 °C for 15 s then brought back to 95 °C. Dissociation curves confirmed single peaks of the predicted size without primer dimerization. All assays included three technical replicates with template-free and non-RT as controls; and the relative expression was calculated using the 2^- △△^ Ct method (Livak and Schmittgen, 2001), normalized to the whitefly 60S ribosomal protein L13a (*RPL13*) gene. Mean Ct value of three technical replicates was calculated per sample.

### 2.9 Identification and phylogenetic analysis of candidate whitefly dsRNase genes

To obtain an initial set of candidate nucleases in *B. tabaci*, the translated sequence of the non-specific nuclease *Bombyx mori*, which has been demonstrated to degrade dsRNA and suppress RNAi (Arimatsu et al., 2007, Liu et al., 2012b), was BLASTed (*E* value < 1.0 e^−10^) against the translated RefSeq genes in the *B. tabaci* genome (Chen et al., 2016). The resultant *B. tabaci* genes were analyzed by SignalP (Petersen et al., 2011) and NCBI conserved domain database for signal peptide and conserved domain, respectively. For analysis of gene phylogenies, a neighboring-joining tree was constructed of the conserved DNA/RNA non-specific nuclease domain amino acid sequences (MEGA 6.06).

### 2.10 Tomato plant grafting

For each graft, a scion with few mature leaves from 5-6-week-old wild-type tomato was cut and inserted to a transgenic dsRNA plant of the same age. The graft site was fastened with Parafilm; and the plant was covered with a plastic bag and kept in darkness at 25 °C for 48 h. Lighting was gradually increased over the next 3–4 days, and the bag was removed at day-7. Wildtype scions were grafted to two GAS1:ds-*GFP* and two AtSUC2:ds-*GFP* plants. The plants were harvested 3 weeks after grafting for RNA isolation and northern blotting analysis of ds-*GFP* fragments.

### 2.11 Statistical analysis

All data sets conformed to the expectations of normality by the Anderson Darling test and homogeneity of variance by the Levine and Bartlett tests. They were analyzed by one-way ANOVA with Fisher's LSD *post hoc* test. Statistical analyses were conducted with JMP software (SAS Institute, Miami, USA) and Minitab 17.

## 3 Results

### 3.1 Delivery of plant dsRNA to whiteflies

**Fig. 1:**
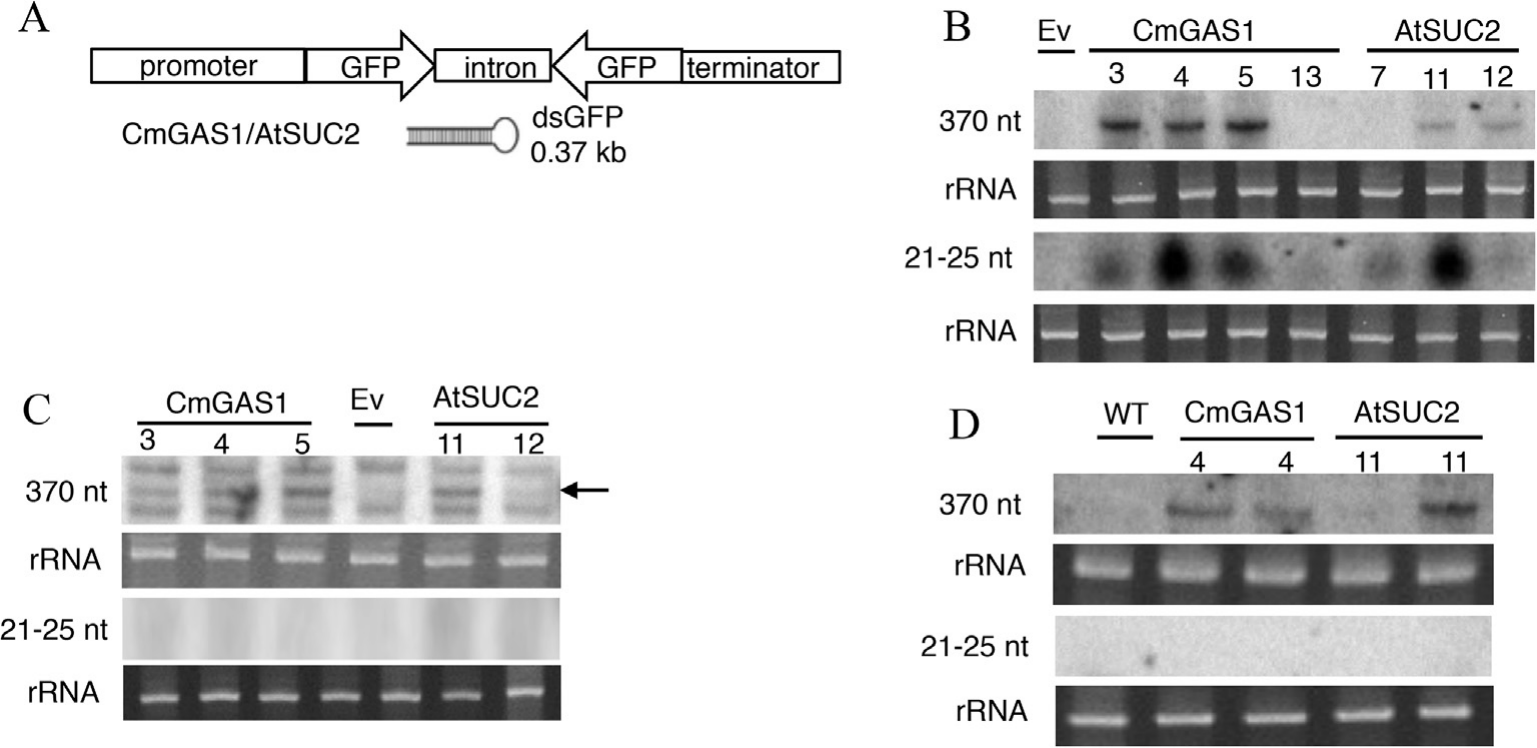
Northern blot analyses of ds-*GFP* in transgenic tomato plants and whiteflies feeding on the plants. (A) Map of transformation vectors for ds-*GFP* expression, designed to produce 370 bp hairpin-ds-*GFP* (hp-ds-*GFP*) under companion cell-specific promoter *CmGAS1* or *AtSUC2*; (B) 370 nt ds-*GFP* (top) and 21–25 nt small RNA (bottom) in the plants. (C) 370 nt ds-*GFP* (horizontal arrowhead) in cohorts of ca. 250 whiteflies that had fed on transgenic plants for two weeks. (D) RNA gel blot analysis of RNA extracted from the scion apex heterografted (*GAS1*:4 and S*UC2*:11) plants. Numbers indicate transgenic tomato lines, with Ev comprising plants transformed with the empty vector, and WT comprising non-transformed tomato plants. Twenty μg total leaf or whitefly low molecular weight RNA (LMW) and high molecular weight RNA (HMW) were separated on 8% denatured urea-acrylamide gel, with the HMW RNA hybridization protocol for full-length ds-*GFP* detection and LMW RNA hybridization protocol for small RNA detection. The rRNA loading control is SybrGold stained 5S rRNA.

The first experiments investigated the fate of dsRNA expressed under companion cell-specific promoters *CmGAS1* from melon, *Cucumis melo* (expressed in companion cells of minor veins of the leaf) and *AtSUC2* from *Arabidopsis thaliana* (expressed in all companion cells) in transgenic lines. The 370 bp ds-*GFP* construct (Fig. 1A) used in these experiments had no sequence homology to tomato or whitefly genes, to ensure that processing of the dsRNA would not be altered by sequence-specific effects of the dsRNA on gene expression in the plant or insect.

**Fig. S1:**
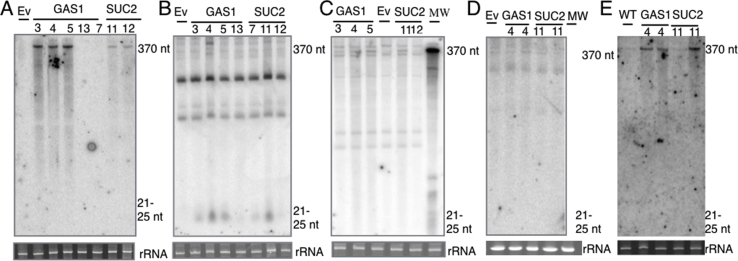
Northern blot analyses of ds-*GFP* in transgenic tomato plants and whiteflies feeding on the plants. (A) Plant RNA with HMW RNA extraction and HMW RNA hybridization protocols. (B) Plant RNA with LMW RNA extraction and LMW RNA hybridization protocols. (C) Whitefly with HMW RNA extraction and HMW RNA hybridization protocols. (D) Whitefly with LMW RNA extraction and LMW RNA hybridization protocols. (E) RNA extracted from the scion apex heterografted (*GAS*:4 and S*UC2*:11) plants. RNA was extracted from tomato leaves (A,B,E) and from ca. 250 adult whiteflies that had fed on transgenic plants for two weeks (C,D), and the samples (20 μg RNA per lane) were separated on 8% denatured urea-acrylamide gel. Numbers indicate transgenic tomato lines, with Ev, the negative control, comprising tomato plants transformed with the empty vector. WT, wild-type tomato, MW, molecular weight. The rRNA loading control is SybrGold stained 5S rRNA. The bands of molecular weight intermediate between 370 nt GFP and candidate sRNAs (21–25 nt) were interpreted as non-specific binding of plant and whitefly RNA to the GFP probe because they were detected in both the experimental samples and Ev controls. The LMW RNA extraction protocol (B, D) reduced, but did not exclude HMW RNA, particularly yielding multiple bands >> 21–25 bp in the plant samples (B) that were detectable with the low stringency of the LMW hybridization protocol required to visualize 21–25 bp bands.

Northern blotting of ds-*GFP* fragments expressed under the *CmGAS1* and *AtSUC2* promoters in 5-6-week-old transgenic tomato lines yielded a band at ca. 21–25 nt in all lines and at 370 nt in most lines (Fig. 1B). These results are compatible with the interpretation that plant Dicer enzyme(s) cleaved the full-length ds-*GFP* into sRNA(s). (Our methods could not discriminate whether the sRNA product was one or multiple molecules in the range 21–25 nt.) Smearing of the signal down the gel, indicative of non-specific degradation of ds-*GFP*, was very limited in northern blots of plant RNA (Figs. S1A and B, whole gel picture of Fig. 1B).

The northern blots for the whiteflies that had fed on the ds-*GFP* plants bore either two or three bands in the region of the predicted 370 nt ds-*GFP* band (Fig. S1C). The middle of these three bands was the closest match to the 370 nt ds-*GFP* probe (Fig. S1C and Fig. 1C) and was detected in the whiteflies feeding from ds-*GFP* plants but not the empty vector control plant. The other two bands were evident in all the samples including the empty vector control, indicative of non-specific binding of whitefly RNA to the GFP probe. These results suggest that the whiteflies feeding on the ds-*GFP* plants are ingesting ds-*GFP.* The blots for the whiteflies bore smeared signal over an extended range of the gel, indicative of non-specific degradation, and no detectable sRNA signal. (Fig. S1C&S1D, whole gel picture of Fig. 1C).

We postulated two alternative explanations for the apparent absence of *GFP* sRNA in the whiteflies: that the sRNA, first, is not phloem-mobile and consequently not ingested by the whiteflies; and, second, it is ingested but degraded in the whitefly. To test for the phloem mobility of the full-length and sRNA, control scions were grafted onto transgenic plants expressing ds-*GFP* under *CmGAS1* or *AtSUC2* promoters, with homografts onto wild-type (WT) plants as negative control. Three weeks after grafting, entire scion apices were excised and processed for *GFP*-RNA by northern blotting. The 370-nt band but not the sRNA band, was detected (Fig. 1D and Fig. S1E). These data indicate that the processed sRNA derived from the ds-*GFP* is not phloem-mobile, and therefore not available to the whiteflies feeding on phloem sap from the transgenic plants.

### 3.2 dsRNA degradation in the whiteflies

**Fig. 2:**
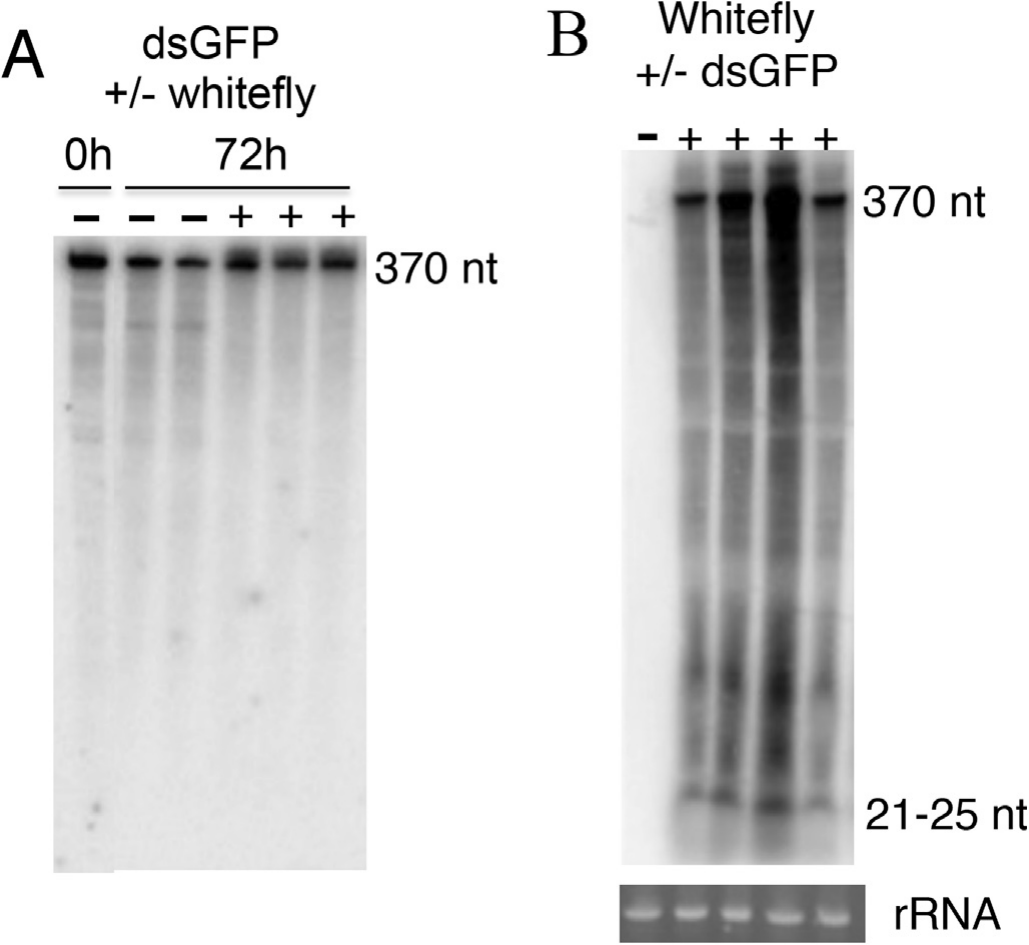
Northern blot analyses of ds-*GFP* administered to whiteflies via artificial diet. (A) Northern blot of diet supplemented with 1 μg ds-*GFP* μl^−1^ and harvested immediately (0 h) and after incubation for 72 h without whiteflies (−) or with whiteflies (+), with 100 ng RNA loaded per well. (B) Northern blot of whiteflies that had fed on two diets containing ds-*GFP* (+) or ds-*GFP*-free diets (−), with 20 μg whitefly total RNA loaded in each lane and Sybr-Gold stained rRNA as loading control. The LMW RNA hybridization protocol was used for both blots.

To investigate the fate of dsRNA molecules in the whiteflies further, adult insects were administered ds-*GFP* via artificial diet. For these experiments, we used the LMW hybridization protocol to visualize the total RNA from diet and whitefly because this protocol enabled us to detect LMW RNAs at low abundance as well as HMW RNA. The 370 nt ds-*GFP* was recovered in northern blots of both the diet on which the whiteflies had fed and diet without whiteflies (Fig. 2A). Parallel analysis of the whiteflies yielded the 370 nt *GFP* band (as in the diet), a smear of signal along the length of the gel, indicative of non-specific degradation, and a sRNA band at *ca.* 21 nt (Fig. 2B). These data suggest that extra-oral degradation of dsRNA, as mediated by salivary secretions in *Lygus* bugs (Allen and Walker, 2012), is not substantial in the whitefly, but that an appreciable portion of ds-*GFP* ingested by the whiteflies is subjected to non-specific degradation within the insect body. We hypothesized that dsRNA ingested by whiteflies is subjected to non-specific degradation in the gut lumen, restricting the availability of dsRNA molecules for uptake by cells of the gut epithelium and intracellular processing by the RNAi machinery. To test this hypothesis, we applied phylogenetic methods to identify candidate nuclease genes in the *B. tabaci* genome.

### 3.3 Phylogenetic analysis of candidate dsRNase genes in the Bemisia tabaci genome

**Fig. 3:**
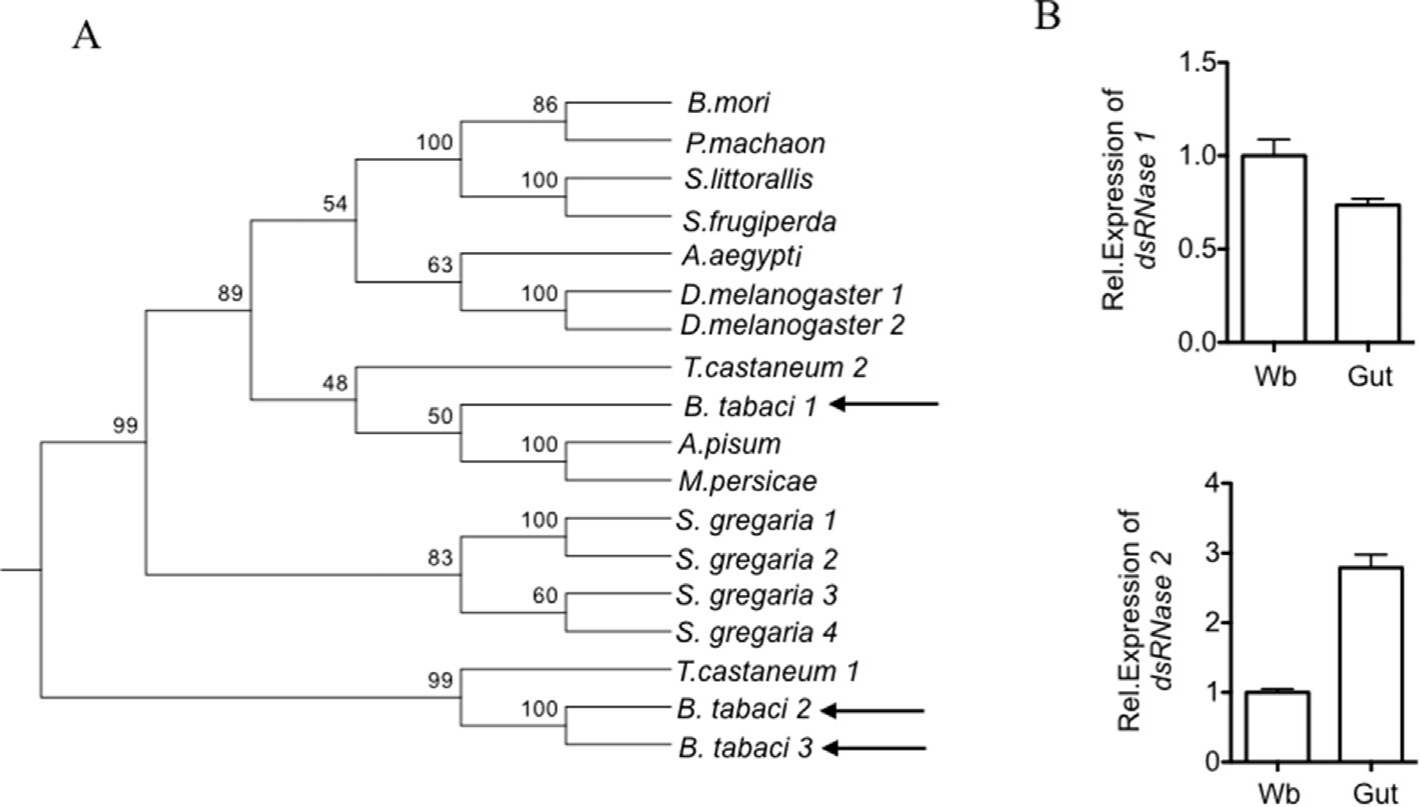
The *dsRNase* genes of *Bemisia tabaci*. (A) Neighbor-joining phylogenetic tree constructed using the protein sequence of the conserved DNA/RNA non-specific nuclease domain of insect *dsRNase* genes. The numbers at the branches indicate the %bootstrap support, based on the frequency of the clusters for 1000 bootstraps. The *d*sRNase sequences were: *Bombyx mori* 2 (NP_001091744.1), *Papilio machaon* (XP_014355571.1), *Spodoptera littorallis* (CAR92522.1), *Spodoptera frugiperda* (CAR92521.1), *Aedes aegypti* (XP_001648469.1), *Drosophila melanogaster* 1 (NM_140821.4), *D. melanogaster* 2 (NP_649078.1, CG3819), *Tribolium castaneum* 1 (XP_973587.1), *T. castaneum* 2 (XP_970494.1), *Schistocerca gregaria* 1 (KJ135008), *S. gregaria* 2 (KJ135009), *S. gregaria* 3 (KJ135010), *S. gregaria* 4 (KJ135011), *Acyrthosiphon pisum* (ACYPI008471), *Myzus persicae* (MYZPE13164_0_v1.0_000125730.4_pep) and *Bemisia tabaci* 1(KX390872), *B. tabaci* 2 (KX390873) and *B. tabaci* 3 (Unigene11878_BT_Q_SG_ZJU). (B) qRT-PCR analysis of the expression of *BtdsRNase-1* (top) and *BtdsRNase-2* (bottom) in dissected guts of *B. tabaci*, relative to the whole body (Wb). Mean ± s. e. from 3 replicates are shown.

Our strategy to identify candidate nuclease(s) in the *B. tabaci* genome was to identify orthologs of a *Bombyx mori* DNA/RNA non-specific nuclease gene (*BmdsRNase*) that has been validated experimentally to cleave dsRNA and reduce the efficacy of RNAi (Liu et al., 2012b). The *B. mori dsRNase* was BLASTed (*E* value < 1.0 e^−10^) against transcriptome databases of the whole body, salivary gland and gut of *B. tabaci*, yielding three *B. tabaci* sequences (*dsRNase1, dsRNase2, dsRNase3*) with a single DNA/RNA non-specific nuclease domain (NCBI conserved domain database) and a predicted signal peptide (SignalP). A phylogenetic tree constructed by the neighbor-joining method using the amino acid sequence of the conserved DNA/RNA non-specific nuclease domain from multiple insect species aligned the *B. tabaci dsRNase1* with aphid nucleases with moderate bootstrap support, and *dsRNase2* and *dsRNase3* with nuclease-1 of *Tribolium castaneum*, with excellent bootstrap support (Fig. 3A).

We amplified part of the predicted full-length cDNA sequences of *dsRNase1* and *dsRNase2* from whole body and gut cDNA libraries of adult *B. tabaci*, but failed to amplify *dsRNase3*. Complementary searches of the *B. tabaci* whole body, gut and salivary gland transcriptome databases (Su et al., 2012, Wang et al., 2010, Wang et al., 2012) yielded a few *dsRNase3* reads only in the salivary gland transcriptome, suggesting that this gene is weakly expressed in the adult insects. Validating these transcriptome data, qRT-PCR analysis of the *B. tabaci* used in this study confirmed that *dsRNase1* and *dsRNase2* are expressed, with three-fold enrichment of *dsRNase2* expression in the gut relative to the whole body (Fig. 3B).

### 3.4 Effect of RNAi against dsRNase genes on ds-GFP ingested by the whiteflies

**Fig. 4:**
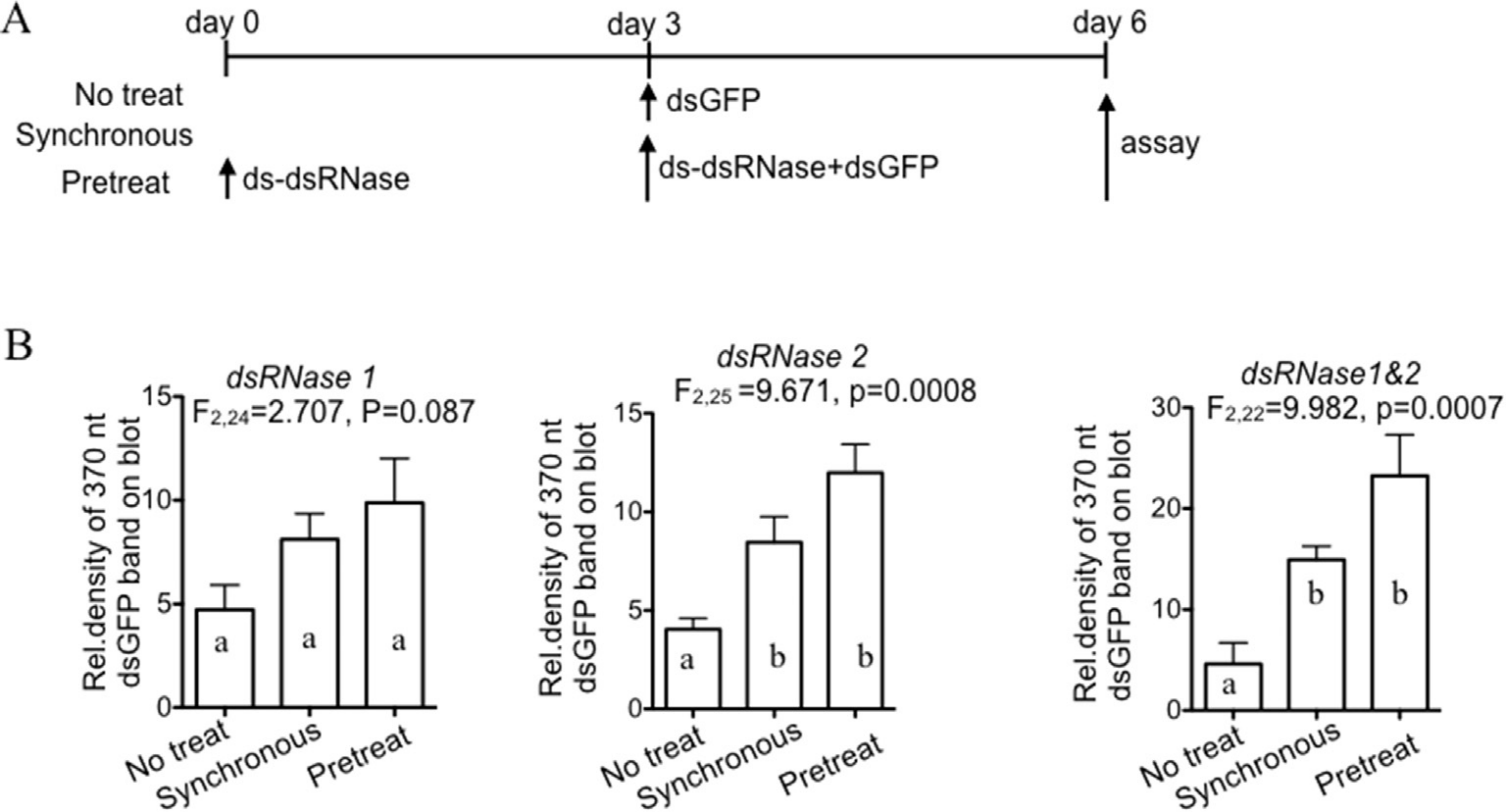
Effect of RNAi against *dsRNase* on the abundance of ds-*GFP* administered orally to whiteflies. (A) Experimental design. 200 ng μl^−1^ of each ds-*dsRNase* and 500 ng μl^−1^ of ds-*GFP* were fed to the whiteflies. (B) Relative density of the 370 nt ds-*GFP* bands on northern blots (mean ± s. e, 3 replicate experiments), with ANOVA results and significantly different treatments by post hoc test indicated by different letters. The northern blots are displayed in Fig. S3.

**Fig. S2:**
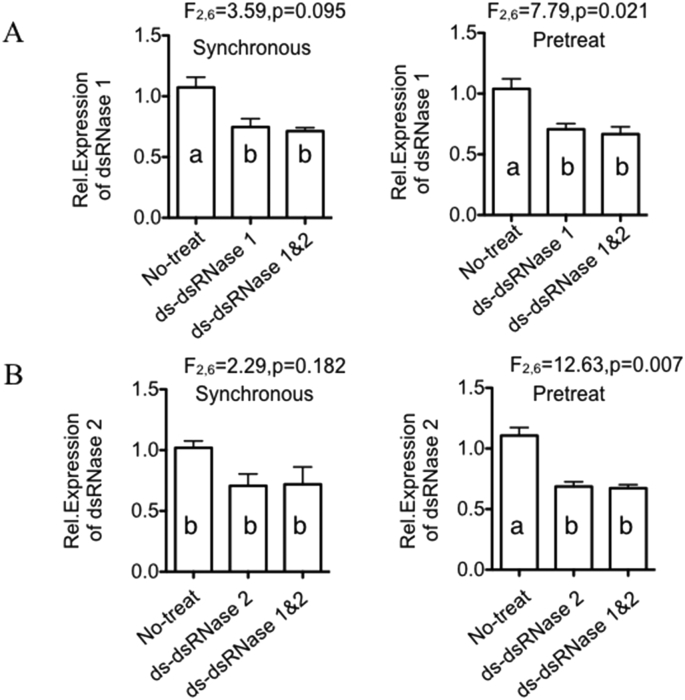
Expression of dsRNase genes in whiteflies administered ds-dsRNase1, ds-dsRNase2 or ds-dsRNase1&2. The experimental design is shown in Fig. 4A. (A) Relative expression level of dsRNase1. (B) Relative expression level of dsRNase2. Gene expression was normalized to RPL13, and mean ± s. e. from 3 replicates are shown. Different letters indicate significantly different treatments by post hoc test following one-way ANOVA.

We next asked whether inhibiting whitefly *dsRNase* genes could protect ds-*GFP* from nonspecific degradation and thus improve RNAi. Enzymatically synthesized dsRNA against *dsRNase1* or *dsRNase2* was fed to the whiteflies either synchronously or 3 days prior to adding ds-*GFP*, with *dsRNase-*free treatment as the control (Fig. 4A). qRT-PCR showed that administration of dsRNA against *dsRNase1* and *dsRNase2* reduced their expression by 25–30% (Fig. S2). Furthermore, northern blots from three independent experiments revealed that the intensity of the full-length ds-*GFP* band and ca. 21 nt GFP was elevated in whiteflies administered dsRNA against the *dsRNase* genes (Fig. S3), and this effect was significant for the whiteflies pretreated with dsRNA against *dsRNase2* and both *dsRNases*, but not *dsRNase1*(Fig. 4B). Because the RNAi-mediated reduction in expression of both dsRNase genes had the greatest protective effect, our subsequent experiments used dsRNA against both genes.

### 3.5 Effect of ds-dsRNase on efficacy of RNAi against whitefly osmoregulation genes

**Fig. S4:**
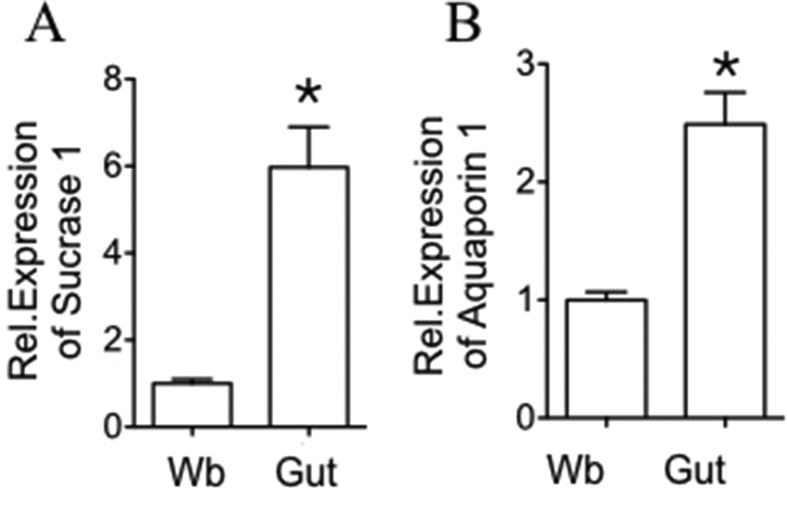
Transcription profiles of SUC1 (A) and AQP1 (B) in the whole body and dissected gut of adult whiteflies. Gene expression was normalized to RPL13. Mean ± s. e. from 3 replicates are shown. * indicate significantly different by Student's t-test.

The demonstration (Fig. 4) that RNAi against the whitefly *dsRNase* genes is protective for ds-*GFP* provided the basis to investigate whether the efficacy of RNAi against whitefly genes of interest can be improved by combination with RNAi against the *dsRNase* genes. Our experiments focused on whitefly genes *AQP1* and *SUC1*, with the predicted function of protecting the insect against osmotic dysfunction (Jing et al., 2016, Mathew et al., 2011). Because these genes have been identified principally by bioinformatics methods, we first conducted qRT-PCR experiments that confirmed the expression of these genes, including their enriched expression in the gut (by 6-fold for *SUC1* and 2.3-fold for *AQP1*), in the adult whiteflies used for our experiments (Fig. S4).

**Fig. 5:**
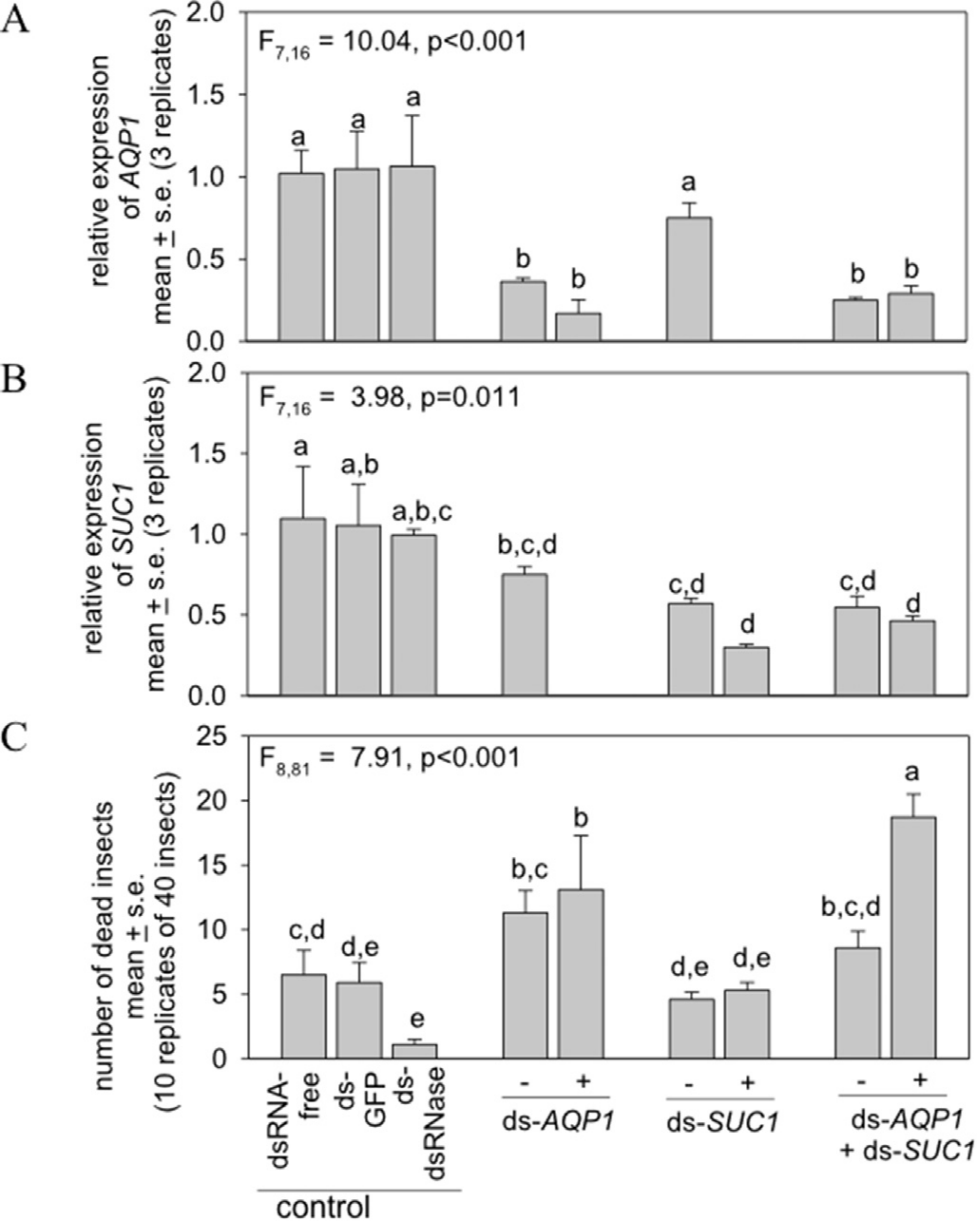
RNAi against osmoregulation genes in adult *B. tabaci* on artificial diet. dsRNA (100 ng μl^−1^ diet) were ds-*GFP*, ds-*dsRNase1&2*, and dsRNA against osmoregulation genes *AQP1* and *SUC1* with (+) or without (−) ds-*dsRNase1&2* administered over 6 days. A&B. qRT-PCR analysis of *AQP1* and *SUC1* expression, respectively, relative to dsRNA-free diet, normalized to *RPL13*. Mean ± s. e, 3 reps. The expression of *SUC1* in insects fed on ds-*AQP1*, and of *AQP1* in insects fed on ds-*SUC1*, was quantified only in the presence of ds-*dsRNase1&2.* C. Number of dead insects (mean ± s. e. for 10 replicates of 40 insects) at day-6. Different letters indicate significantly different treatments by Fisher's LSD test.

**Fig. S5:**
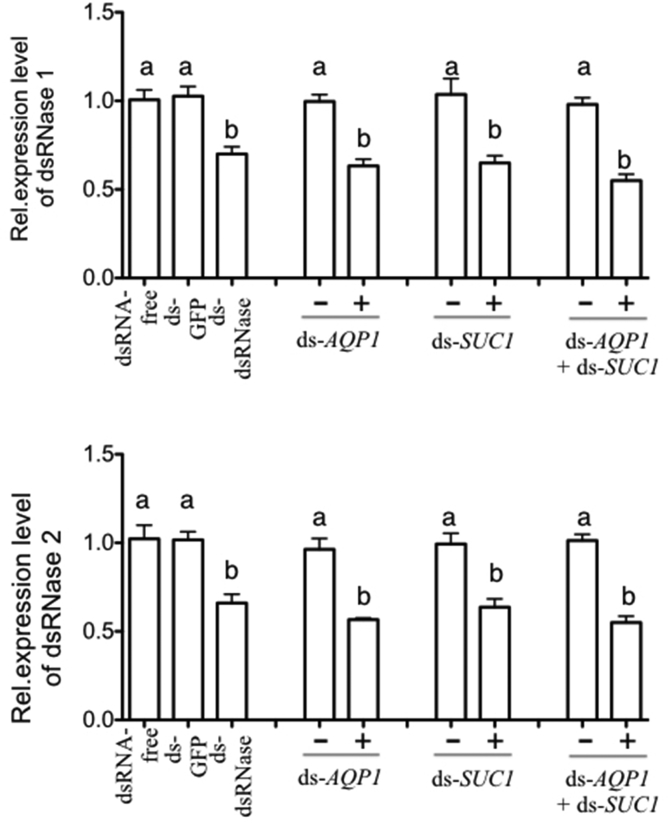
Expression of dsRNase genes in adult whiteflies reared for 6 days on artificial diets supplemented with ds-dsRNase1&2 (+), with ds-dsRNase1&2-free diets (−) as control. (A) Relative expression level of dsRNase1 (B) Relative expression level of dsRNase2. Gene expression was normalized to RPL13, and mean ± s. e. from 3 replicates are shown. Different letters indicate significantly different treatments by post hoc test following one-way ANOVA.

We delivered the dsRNAs to adult whiteflies via artificial diet over a time-course of 6 days. The experimental treatments were dsRNA against the whitefly *AQP1* and *SUC1*, either separately or in combination, and with or without the dsRNA against *dsRNase1* and *dsRNase2*. Surviving insects on day-6 were used for gene expression analysis by qRT-PCR. The ds-*dsRNase* treatments reduced expression of the cognate *dsRNase* genes by 30–35% (Fig. S5) but, when administered as the sole dsRNA, did not affect expression of the target osmoregulation genes (Fig. 5A and B). Expression of *AQP1* and *SUC1* was reduced in whiteflies feeding from diets containing ds-*AQP1* and *dsSUC1*, respectively, relative to the controls (diets with ds-*GFP* or ds-*dsRNase1&2*, and dsRNA-free diets) and these effects were statistically significant apart from ds-*SUC1* without ds-*dsRNase1&2*. Inclusion of ds-*RNase1&2* in the diet reduced mean expression of *AQP1* and *SUC1* relative to diets without ds-*RNase1&2* but the differences were not statistically significant. However, this comparison may not be a reliable indicator of the efficacy of suppression *dsRNase1&2* expression. Specifically, *AQP1* and *SUC1* are expressed in whitefly organs other than the gut, but the effect of ds-*RNase1&2* on the RNAi-suppression of their expression may be greater in the gut than other organs. This pattern could result from limited systemic spread of RNAi (a likely constraint on the efficacy of RNAi in various insects (Scott et al., 2013)) with the consequence that our quantification of transcript abundance in the whole insect bodies may have underestimated the impact of ds-*RNase1&2* on expression of *AQP1* and *SUC1* in the whitefly gut.

The whiteflies in the two treatments containing both ds-*AQP1* and ds-*dsRNase1&2* displayed elevated mortality, relative to the control diets (ds-*GFP* and dsRNA-free). In the absence of ds-*AQP1*, ds-*SUC1* had no discernible effect on whitefly mortality, but functioned synergistically with ds-*AQP1* in the presence of ds-*dsRNase1&2* to yield mortality approaching 50% and significantly greater than all other treatments.

## 4 Discussion

The development of effective RNAi against plant sap-feeding insects cannot be achieved by trial-and-error. It requires a rational understanding of the fate of dsRNA molecules, in both the plant tissues and in the feeding insects. This study demonstrates the complexity of these processes, and identifies a strategy that improves the efficacy of RNAi against the globally-important insect pest, the whitefly *B. tabaci*.

A critical factor determining the efficacy of *in planta* RNAi against phloem-feeding insects is the molecular identity of the phloem-mobile dsRNA molecules. It is inherently difficult to identify phloem-mobile compounds with certainty. In particular, some widely-used methods for direct sampling of the phloem contents of the plant sieve elements (incisions and EDTA exudation) are now recognized to produce material that is contaminated by the contents of other plant cells (Liu et al., 2012a, Zhang et al., 2012). However, reliable information about phloem-mobile RNA molecules can be obtained from the RNA content of both wild-type tissue grafted onto transgenic plants and phloem-feeding insects; and both of these approaches have been used previously (Dunoyer et al., 2010, Melnyk et al., 2011a, Yoo et al., 2004). When these two methods were applied in this study to stable transgenic tomato containing ds-*GFP* under two alternative phloem-specific promoters, they yielded the full-length 370 nt dsRNA, but not sRNA in the 20–25 nt range. This result cannot be attributed to technical difficulties in detecting sRNA because both full-length dsRNA and sRNA were detected in bulk transgenic leaf samples of the same total RNA content. Detailed interpretation of this result requires consideration of the structural relationships among plant cells. Specifically, the ds-*GFP* synthesis is restricted to phloem companion cells, with predicted delivery via large diameter plasmodesmata to the sieve elements and via more sparse and narrow-diameter plasmodesmata to non-vascular plant cells that have little plasmodesmatal contact with the sieve elements (Kempers et al., 1993). Thus, the recovery of dsRNA, but not sRNA, in the sieve elements can, in principle, be attributed to multiple processes: dsRNA processing in the non-vascular tissue and not the companion cells; a selective barrier reducing the delivery of sRNA (but not dsRNA) from the companion cells to the sieve elements; degradation of sRNA in the sieve elements; and the efficient re-uptake of sRNA from sieve elements back into companion cells. Further research is required to establish the relative importance of these different processes. Interestingly, this study adds to the diversity of dsRNA products reported in phloem sap, including short single-stranded RNA in cucurbits and a legume (castor bean) (Yoo et al., 2004) and 21 nt or 24 nt siRNA in *Arabidopsis* (Dunoyer et al., 2010, Melnyk et al., 2011a). Although the basis for these different results is uncertain because the various studies use both different methods and plant systems, one possible interpretation is that the phloem-mobile compounds vary with plant species. Generally, RNAi in insects is more effective with long dsRNA molecules than sRNAs (either single- or double-stranded) (see Introduction), suggesting that the tomato transgenic lines containing dsRNA under phloem-specific promoters, as used in this study, have the potential for effective RNAi against whiteflies.

Further insight into the delivery of *in planta* RNAi to phloem-feeding insects came from our comparison of the ds-*GFP* products in whiteflies feeding from ds-*GFP* transgenic plants and ds-*GFP*-supplemented artificial diet. Specifically, sRNA was detected in insects fed on ds-*GFP* via the diet but not the plant. A parsimonious interpretation of this discrepancy is that non-specific nuclease activity in the insect gut degraded the dsRNA in the phloem sap, quantitatively preventing dsRNA delivery to gut cells, where siRNA is generated by the cytoplasmic RNAi machinery; but that the high concentrations of dsRNA in the artificial diets saturated the gut nuclease activity, such that a proportion of the ingested dsRNA was translocated to gut cells, yielding detectable sRNA in the insect.

It is becoming increasingly evident from this and other studies that non-specific nucleases in the gut lumen or saliva are a key factor limiting the efficacy of orally-delivered dsRNA in insects (Allen and Walker, 2012, Arimatsu et al., 2007, Liu et al., 2012b, Spit et al., 2017, Wang et al., 2016, Wynant et al., 2014). The cognate nuclease gene family is conserved across insects and some crustaceans, and has also been reported in one viral genome (the shrimp white spot syndrome virus in the Nimaviridae) but lacks homologs in other animals, raising the possibility that it was acquired horizontally in the common ancestor of insects and crustaceans (Wynant et al., 2014). Although these genes likely have an anti-viral function, as indicated by the increased expression of the *dsRNase* gene in *Bombyx mori* infected with cytoplasmic polyhedrosis virus (Wu et al., 2009), they are also strong candidates as suppressors of orally-delivered RNAi. Consistent with this interpretation, hemolymph injections of dsRNA against the *dsRNase* genes resulted in reduced transcript abundance of *dsRNase* genes and dsRNase enzymatic activity in the midgut of in the locust *Schistocerca gregaria* (Wynant et al., 2014).

This study extends the application of RNAi in whiteflies to test whether RNAi-mediated suppression of *dsRNase* genes results in enhanced efficacy of RNAi against other insect genes, specifically the two predicted osmoregulation genes, *AQP1* and *SUC1*, identified previously by Jing et al. (2016) and Mathew et al. (2011). As predicted, orally-delivered ds-*dsRNase1&2* both protected ds-*GFP* from non-specific degradation and increased the efficacy of RNAi against osmoregulation genes, as quantified by gene expression and survivorship of insects administered dsRNA via artificial diet. Interestingly, relatively modest suppression of expression of the *dsRNase* genes resulted in substantial protection of orally-delivered *dsGFP* (Fig. 4). We hypothesize that this effect may result from a very short half-life of the nuclease proteins coded by the *dsRNase* genes (i.e. these proteins have a high turnover rate), resulting in a large reduction in nuclease activity for relatively small changes in transcript abundance. More generally, our results are paralleled by the recent demonstration that the efficacy of RNAi against the Colorado potato beetle *Leptinotarsa decemlineata* is also enhanced by co-administration of dsRNA against gut nuclease genes (Spit et al., 2017). These data suggest that the efficacy of RNAi in various insects (Scott et al., 2013, Terenius et al., 2011, Wang et al., 2016) may be significantly improved by RNAi-mediated suppression of *dsRNase* genes, and potentially other RNAi-suppressors.

It has been argued that the efficacy and durability of RNAi for insect pest control can be enhanced by stacking dsRNA constructs against multiple targets in the pest species (Scott et al., 2013, Tzin et al., 2015). This strategy is likely particularly valuable where the target genes have unrelated molecular function but linked physiological function because the incomplete suppression of multiple contributing gene functions can combine synergistically to mediate physiological collapse. This effect is the most likely explanation for the enhanced efficacy of stacking dsRNA constructs against multiple osmoregulation genes in the phloem-feeding aphid *Myzus persicae* and psyllid *Bacteriocera cockerelli* (Tzin et al., 2015). In our hands, however, the performance of the whitefly *B. tabaci* is not significantly reduced by RNAi against osmoregulation genes, either individually or stacked (this study; unpub results); and RNAi efficacy is dependent on co-targeting of the *dsRNase* genes (this study).

Also pertinent to our understanding of the efficacy of RNAi in whiteflies are the results of Raza et al. (2016), who transformed tobacco plants with dsRNA against the *B. tabaci AQP1* and *SUC2* genes, obtaining 80->98% expression knockdown of the target genes and 60–80% insect mortality over 6 days. Further research is required to identify the factors contributing to the differences between the two studies, potentially including variation in the expression of gut nuclease genes among different genotypes of *B. tabaci*, and differences in the identity of phloem-mobile dsRNA molecules between tobacco used by Raza et al. (2016) and tomato used in this study. In addition, Raza et al. (2016) used a dsRNA construct against *SUC2*, which lacks a signal peptide sequence (Jing et al., 2016). The SUC2 protein likely has an intracellular function, as distinct from SUC1, used in this study, which has a signal peptide permitting export to the gut lumen and osmoregulatory function (Jing et al., 2016).

In conclusion, this study has provided proof-of-principle that the efficacy of RNAi against the whitefly *B. tabaci* can be enhanced by the dual strategies of, first, stacking dsRNA against multiple genes with related physiological roles but distinct molecular functions and, second, “using RNAi to suppress suppressors of RNAi”. These approaches create wider opportunities for RNAi as a control strategy against *B. tabaci*, which is a globally important crop pest, and offer a template for comparable strategies against other insect pests with poor or variable RNAi efficacy.

## Contributions of authors

YL: conducted the experiments using ds-*GFP* and osmoregulation genes on artificial diets, and phylogenetic analysis of *dsRNase* genes. QC: designed and generated the constructs for plant transgenesis. JL and SHC: conducted qPCR experiments. JVE: conducted the plant transformations. YL, JL, SHC, JVE, RT and AED: devised the experimental plan, interpreted the results and wrote the manuscript. All coauthors have commented on drafts of the manuscript, and have read and agree to the submitted version.

## Appendix A Supplementary data

Fig. S3 Northern blots of ds-GFP fragment following treatment with ds-dsRNase1, ds-dsRNase2 and ds-dsRNase1&2, with SybrGold–stained 5S rRNA as loading control. The LMW RNA hybridization protocol was used for all blots. The exposure time for each blot was optimized independently, to ensure that none of the bands on each blot was saturated and so ensure the reliability of the data in Fig. 4B. Biological variability and the criteria for optimizing exposure time (above) contribute to the among-experiment variation. No: no treatment; Syn: synchronous treatment with ds-GFP and ds-RNase; Pre: pretreatment with ds-dsRNase (see Fig. 4A for details). Fig. S3

Table S1 Primers. Table S1
