# Supplementary material for: Towards an understanding of the molecular basis of effective RNAi against a global insect pest, the whitefly Bemisia tabaci
Source: Insect Biochem Mol Biol. 2017 Sep;88:21–9. doi: 10.1016/j.ibmb.2017.07.005 (PMC5595799; doi:10.1016/j.ibmb.2017.07.005)
Supplement: Fig. S3 — Northern blots of ds-GFP fragment following treatment with ds-dsRNase1, ds-dsRNase2 and ds-dsRNase1&2, with SybrGold–stained 5S rRNA as loading control. The LMW RNA hybridization protocol was used for all blots. The exposure time for each blot was optimized independently, to ensure that none of the bands on each blot was saturated and so ensure the reliability of the data in Fig. 4B. Biological variability and the criteria for optimizing exposure time (above) contribute to the among-experiment variation. No: no treatment; Syn: synchronous treatment with ds-GFP and ds-RNase; Pre: pretreatment with ds-dsRNase (see Fig. 4A for details). [file mmc2.pdf]

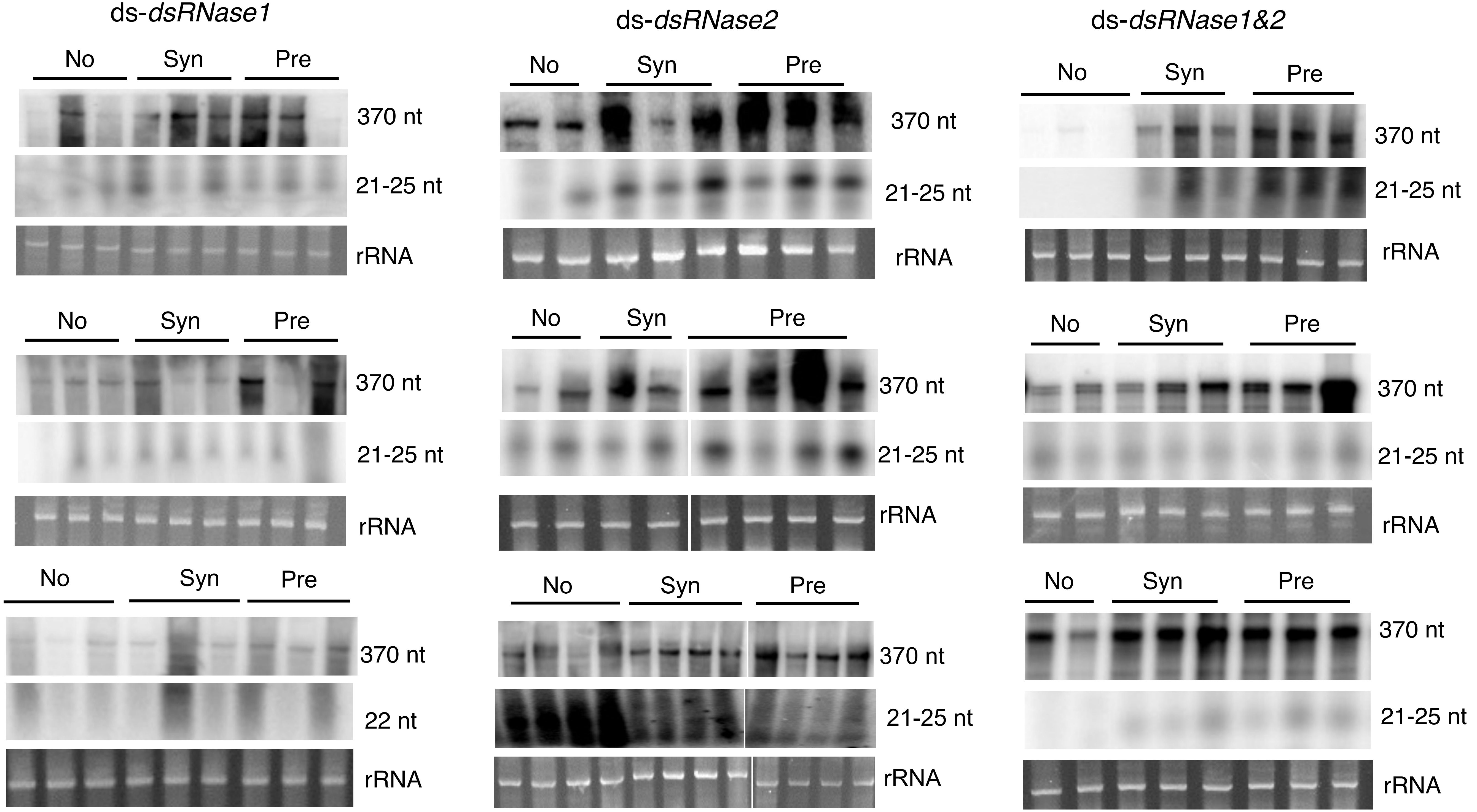

ds-*dsRNase1*
ds-*dsRNase2*
ds-*dsRNase1&2*
No
Syn
Pre
370 nt
21-25 nt
22 nt
rRNA
