# Supplementary material for: Towards an understanding of the molecular basis of effective RNAi against a global insect pest, the whitefly Bemisia tabaci
Source: Insect Biochem Mol Biol. 2017 Sep;88:21–9. doi: 10.1016/j.ibmb.2017.07.005 (PMC5595799; doi:10.1016/j.ibmb.2017.07.005)
Supplement: Table S1 — Primers. [file mmc1.docx]

Table S1 Primers

| Gene | Forward primer 5'-3' | Reverse primer 5'-3' |
| --- | --- | --- |
| (a) primers for gene verification | | |
| *dsRNase1* | CGCTGATGAAACCGGAAATG | GCTTGTGGCACTCTTGTTATG |
| *dsRNase2* | CGTTGGCGCAGTTTGTAAAG | CCACTCGCATTTGAGAGGAA |
| *SUC1* | GATCTAGCTTGGTGGGAGAAAG | TGTATGTCGGGTGGTCTTTG |
| *AQP1* | CATAGTTGCTGAGTTCGTAGGG | GCTTTGAAAGTGATGGCGTAAA |
| (b) primers for dsRNA synthesis (with T7 promoter sequence: TAATACGACTCACTATAGG) | | |
| *dsGFP* | ATGGGTAAAGGAGAAGAAC | ATCCTGTTGACGAGGGTGTC |
| *dsRNase1* | TACGAACATTAACGGGAACGACGG | GGTATAGCTGGGTTCACCTTCAGTG |
| *dsRNase2* | CAGTGGCAAATCATCAATGCG | ATGTGGAGATTTATTTACAGCCAG |
| *SUC1* | GATCTAGCTTGGTGGGAGAAAGG | GGGGAGCGAAAAATCGGAGA |
| *AQP1* | TTCGTAGGGACTTTGCTGTTAG | CGGATTGATGTGACAACCACTAA |
| (c) primers for qRT-PCR | | |
| *dsRNase1* | GAAACTCGCTCCTCTTGTAGTT | TGTCTGCTCTTCCTGTCTTATTC |
| *dsRNase2* | CGAGTGCACGAGTAGTGTAAA | CACACCCACATCAGAGGTAAA |
| *SUC1* | GACTGGTATGCTGCTCTCCC | CATCTGAAGCATGTGCAGCC |
| *AQP1* | GGAGCCATCTGTGGAGCAAT | AGTGCTTCTATCGCCACACC |
| *RPL13* | TAAATTCGACTCGACTCACGGT | CTCCACCACATACTCGGCTC |
| (d) Primers for identification of positive transformants | | |
| *GFP* ATGGGTAAAGGAGAAGAAC ATCCTGTTGACGAGGGTGTC | | |

_____________________________________________________________________________________________
